# Cutaneous vasculitis in children: A nationwide epidemiological study in Spain

**DOI:** 10.12688/f1000research.12372.1

**Published:** 2017-08-21

**Authors:** Leyre Riancho-Zarrabeitia, Ana Santurtún

**Affiliations:** 1 Department of Rheumatology, Sierrallana Hospital , Torrelavega, 39300, Spain; 2 Department of Physiology and Pharmacology, Legal Medicine Unit, University of Cantabria, Santander, 39011, Spain

**Keywords:** Cutaneous vasculitis, IgA vasculitis, Children

## Abstract

**Background**: Cutaneous vasculitis (CV) are a complex group of conditions in children, of which IgA vasculitis (IgAV) is the most common. The objectives of the current study are to describe the incidence of CV in Spain and to analyze the temporal trend in the last 11 years, as well as it seasonal distribution.

**Methods:** Hospital discharges of patients aged 0-18 years with a diagnosis consistent with CV in Spain from 2005 to 2015 were collected from the Spanish National Institute of Statistics (INE) databases.

**Results**: A total of 7304 patients from January 2005 to December 2015 were included; 6991 patients (95%) had a diagnosis of IgAV. The yearly incidence in the whole group was 7.7 per 100,000. Mean age at diagnoses was 6±3 years and 52% were male. The highest rate of admissions was found in the 5-9 year-old group, followed by those with 0-4 years of age (15.7 and 9.0 admissions per 100.000, respectively). Admissions due to CV followed an annual cyclic pattern, with the highest number of daily admissions during fall and winter months and the lowest number in summer months. There was an overall downwards trend of the number of hospital admissions during the period of study, in both males and females (p=0.01).

**Conclusions**: We have estimated an incidence of a 7.7 cases per 100,000 CV in children in Spain. CV-related hospitalization rates have a marked seasonal pattern, with a peak in fall and winter and a nadir in summer months. Children between 5 and 9 years of age are most frequently affected. There is a decreasing trend in CV-related hospitalization, the causes of which should be further assessed.

## Introduction

Cutaneous vasculitis (CV) are a complex group of conditions in children. The most common are IgA vasculitis (IgAV) (formerly known as Schonlein-Henoch purpura, (SHP)), which represents more than half of the cases, followed by cutaneous small-vessel vasculitis (formerly known as hypersensitivity vasculitis). Other disorders, such as urticarial vasculitis or ANCA associated vasculitis are poorly represented in children ^1^. The global incidence is not known ^2^, while incidence of IgAV in children range from 3–26.7 per 100,000 ^3^. Symptoms vary from a cutaneous-limited disorder to a systemic disease, and the etiology is not fully understood. However in many cases, particularly in IgAV, an external trigger is frequently suspected; IgAV in children has been frequently associated with a preceding upper respiratory infection, but no specific pathogen has been identified. It has also been linked to antibiotics and other medications ^3^. The reported seasonal pattern, with a fall-winter incidence peak, is consistent with the hypothesis of an infectious trigger ^3^.

The aims of our study are to describe the incidence of CV in Spain and to analyze the temporal trend of CV in the last 11 years, as well as it seasonal distribution.

## Methods

Hospital discharges with a diagnosis consistent with CV (International Classification of Diseases ICD-9 codes hypersensitivity angiitis (446.2) and allergic purpura, including SHP (287.0)) in Spain from January 2005 to December 2015 were collected from the Spanish National Institute of Statistics (INE) databases.

We calculated the overall average incidence of admission per 100,000 inhabitants during the 11 years in children (from 0 to 18 years). Moreover, we calculated the annual rate of admission in children, and for the temporary trend calculations, a Kendall’s tau correlation coefficient. Monthly admission rates were compared with Krustal-Wallis test. Statistical analysis were performed with R v2.3.

## Results

**Figure 1.**
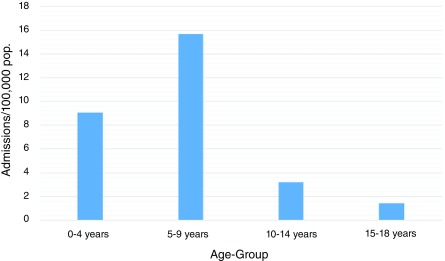
Incidence of cutaneous vasculitis across age groups between January 2005 and December 2015. The highest incidence occurred in children 5–9 years of age.

A total of 7304 patients from 0 to 18 years of age were discharged from January 2005 to December 2015 with a diagnosis of CV. 6991 patients (95%) had a diagnosis of IgAV and 313 had hypersensitivity angiitis. The yearly incidence in the whole group was 7.7 per 100,000. Mean age at diagnosis was 6±3 years and 52% were male, with a male to female ratio of 1.02:1. The highest rate of admissions was found in the 5-9 year-old group, followed by those aged 0-4 years (15.7 and 9.0 admissions per 100.000, respectively) ( Figure 1).

**Figure 2.**
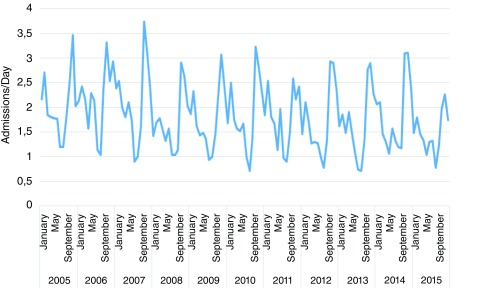
Monthly incidence of cutaneous vasculitis during the period of study (January 2005–December 2015). A cyclic pattern was revealed, with a peak during fall and winters months and a nadir in summer.

**Figure 3.**
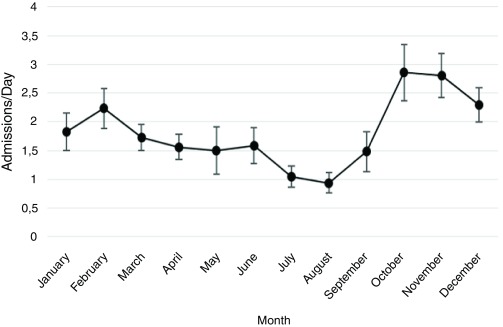
Average (mean and standard deviation) monthly incidence of cutaneous vasculitis during the period of study (January 2005–December 2015). The combined analysis confirms the seasonal pattern throughout the period of study.

Admissions due to CV followed an annual cyclic pattern ( Figure 2), with the highest number of daily admissions during fall and winter months and the lowest number in summer months. This pattern was consistent over the 11 years of study, with a 3-fold increase in the number of daily admissions in October compared with August (p<<0.001; Figure 3).

**Figure 4.**
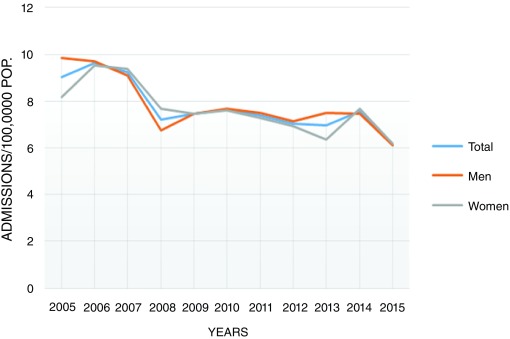
Average annual incidence of cutaneous vasculitis during the study period (January 2005–December 2015). A downwards trend in the overall incidence during the period of study was observed.

The annual analysis showed a downwards trend of the number of hospital admissions during the 11-year period of study, in both men and women (p=0.01; Figure 4).

## Discussion

This is the first population-based study of CV among children in Spain. We report the incidence rate of admissions of children with CV, defined as IgAV and hypersensitivity angiitis, over 11 years.

We estimate a yearly incidence of 7.7 cases per 100.000. Data on incidences rate on CV are scarce, while previous series on IgAV have reported incidences that range from 6.1 in the Dutch population ^4^ to 20.4 in the United Kingdom ^5^. Most published series report incidences between 10 and 20 cases per 100.000, with some discrepancies probably due to the heterogeneity of the criteria used and also by the source of identification of cases (those based exclusively in hospital discharge data fail to identify children not referred to the hospital). The incidence we report in Spanish children keeps in line with previous literature, being in the lower part of the range. Our estimates are based on hospitalized cases, which might somewhat underestimate true incidence. However, we feel our estimated incidence should be close to the true incidence, as most cases are attended to at a hospital, at least in western countries. This idea is supported by a US study reporting that only 10% of children with IgAV were reported exclusively by primary care physicians ^6^ and by a UK study showing that only 3% of IgAV cases were reported by general practitioners ^5^.

IgAV mainly affects children between 3 and 12 years of age ^3^, with a mean age of 5–6 years in most paediatric series ^6– 8^. A slightly male predominance has been reported, with a male to female ratio of up to 1.8:1 ^5– 7^, while others reported that cases were equally distributed ^8^, or even a subtle female predominance ^9^. In our case, we found a mean age of 6 years with no differences in sex distribution.

We found a remarkable seasonal variation in the frequency of CV. This is in line with other studies showing that IgAV has a seasonal distribution, with a peak during fall and winter and a nadir during summer months ^3, 10^. This keeps in line with a commonly reported upper respiratory infection preceding the onset of the purpura, and a possible infectious trigger for the disease. Moreover, this increase during fall-winter time could also be related with atmospheric circulation patterns, as recently suggested for Kawasaki disease ^11^.

Our annual analysis showed a downwards trend of the number of hospital admissions during the period of study. A similar trend has been reported previously. Okubo *et al*. ^6^ found a significant decreasing trend, with a total annual hospitalization rate of 2.45 per 100,000 children in 2003, falling to 1.89 per 100,000 children in 2012. This decrease could indicate a tendency to treat patients with IgAV in outpatient clinics, but also could reflect a real decrease in the incidence of the disease.

In summary, we have estimated an incidence of a 7.7 cases per 100,000 CV in children in Spain. CV-related hospitalization rates have a marked seasonal pattern, with a peak in fall and winter and a nadir in summer months. Children between 5 to 9 years of age are most frequently affected. There is a decreasing trend in CV-related hospitalization, the cause of which should be further assessed.

## Data availability

Data were downloaded freely from the Spanish National Institute of Statistics (INE) databases: http://www.ine.es/prodyser/microdatos.htm.
