# Supplementary material for: Cutaneous vasculitis in children: A nationwide epidemiological study in Spain
Source: F1000Res. 2017 Aug 21;6:1527. [Version 1] doi: 10.12688/f1000research.12372.1 (PMC6058467; doi:10.12688/f1000research.12372.1)
Supplement: Peer review history

# Peer review: Referee response for version 1

**DOI:** 10.5256/f1000research.13398.r35432

**Published:** 2018-07-20

**Authors:** Robert Micheletti

**Affiliations:** 1 Department of Dermatology and Department of Medicine, University of Pennsylvania, Philadelphia, PA, USA

The manuscript is an interesting epidemiologic study of the incidence of cutaneous vasculitis in children.  In particular, the observation of seasonality of the condition, as well as the estimation of the overall incidence and the declining incidence over time, are informative.

 I do have some questions / criticisms that could perhaps be addressed in the discussion / limitations to make this a stronger paper:

- One limitation is the diagnosis codes used (hypersensitivity angiitis and Henoch-Schonlein purpura).  Are these the only codes?  The best codes?  It seems worth mentioning the limitations of identifying cases in this manner, since not all cases of interest may be identified.  If you are not accurately including other types of cutaneous vasculitis with the "hypersensitivity angiitis" code, perhaps this is just a study of IgA vasculitis alone and should be described in that manner?
- The authors argue that their estimate of hospitalized patients is representative of the true incidence of disease based on studies that primary care and general practitioners only handle a subsegment of cases.  The inference is that most patients, therefore, end up at the hospital.  I don't find this argument particularly persuasive?  What about outpatient dermatology?  Rheumatology?  It is rare for me to manage IgA vasculitis in the hospital, whereas I have many outpatients with the disease.  This limitation should be acknowledged (as the authors do), but probably I would eliminate the arguments related to most patients being hospitalized, etc.
- Additionally, the authors may wish to comment on the changing definition / criteria for IgA vasculitis.  Since the Chapel Hill Consensus Conference nomenclature were revised in 2012 (in the middle of the years analyzed in this study), it is worth considering whether case definition affected frequency of diagnosis.  Historically, children with palpable purpura were just called Henoch-Schonlein purpura without much effort to determine the presence of IgA, etc.  It's possible in recent years some of these patients were classified as having other diagnoses.

I have read this submission. I believe that I have an appropriate level of expertise to confirm that it is of an acceptable scientific standard.

# Peer review: Referee response for version 1

**DOI:** 10.5256/f1000research.13398.r25207

**Published:** 2017-09-19

**Authors:** Javier del Pino-Montes

**Affiliations:** 1 Rheumatology Department, IBSAL, Salamanca University Hospital, Salamanca, Spain

This is an interesting study about the epidemiological data of cutaneous vasculitis in children in Spain. There is little information on this topic. It is common to find data from hospitals or registries but the value of this paper is that the data comes from all over Spain. It would be interesting to know if there are geographical differences,

 Minor revision:

 In the results section and Figure 4, children are analyzed by gender as men and women. Being a pediatric population, it is more consistent to use males and females

 I recommend to accept the paper after minor revisions

I have read this submission. I believe that I have an appropriate level of expertise to confirm that it is of an acceptable scientific standard.
